# Genetic analysis of *APOE* reveals distinct origins and distribution of ancestry-enrichment haplotypes in the Mexican Biobank

**DOI:** 10.1016/j.gendis.2025.101542

**Published:** 2025-01-22

**Authors:** Carmina Barberena-Jonas, Victor Flores-Ocampo, Natalia S. Ogonowski, Stefanie Danielle Piña-Escudero, Ignacio F. Mata, Jennifer S. Yokoyama, Lourdes García-García, Carlos Alberto Aguilar Salinas, ENSANUT consortium, María Teresa Tusié-Luna, Andrés Moreno-Estrada, Miguel E. Rentería

**Affiliations:** a Unidad de Genómica Avanzada (UGA-LANGEBIO), Centro de Investigación y Estudios Avanzados del IPN (Cinvestav), Carretera Irapuato León Km 9.6, Irapuato 36821, Mexico; b School of Biomedical Sciences, Faculty of Medicine, The University of Queensland, Chancellors Pl, St Lucia, Brisbane, QLD 4067, Australia; c Brain & Mental Health Program, QIMR Berghofer Medical Research Institute, 300 Herston Rd, Brisbane, QLD 4006, Australia; d Memory and Aging Center, Department of Neurology, UCSF Weill Institute for Neurosciences, University of California San Francisco, 1651 4th St Suite 212, San Francisco, CA 94158, USA; e Global Brain Health Institute, University of California, 1651 4th St, 3rd, Floor San Francisco, CA 94158, USA; f Genomic Medicine Institute, Lerner Research Institute, Cleveland Clinic, 9500 Euclid Avenue Mailstop NE-50, Cleveland, OH 44106, USA; g Department of Radiology and Biomedical Imaging, University of California, 505 Parnassus Ave, San Francisco, CA 94143, USA; h Instituto Nacional de Salud Pública (INSP), Av. Universidad 655, Santa María Ahuacatitlán, Cuernavaca 62100, Mexico; i Dirección de Investigación. Instituto Nacional de Ciencias Médicas y Nutrición Salvador Zubirán, Vasco de Quiroga 15 Belisario Domínguez Secc 16, Tlalpan, Ciudad de México 14080, Mexico; j Encuesta Nacional en Salud y Nutrición (ENSANUT). Instituto Nacional de Salud Pública (INSP), Av. Universidad 655, Santa María Ahuacatitlán, Cuernavaca 62100, Mexico; k Unidad de Biología Molecular y Medicina Genómica. Instituto de Investigaciones Biomédicas-UNAM/ Instituto Nacional de Ciencias Médicas y Nutrición Salvador Zubirán, Circuito, Mario de La Cueva s/n, C.U., Coyoacán 04510, Ciudad de México 14080, Mexico

The apolipoprotein E (*APOE*) gene, located on chromosome 19, remains the primary genetic factor associated with late-onset Alzheimer's disease.^1^ In European populations, the ε4 haplotype of *APOE*, present in approximately 14% of individuals, significantly increases Alzheimer's disease risk, while the less common ε2 haplotype (∼8%) appears to confer a protective effect.^2^ Despite its significance, *APOE* has not been genetically characterized in Latin American countries, where Alzheimer's disease-related dementia disproportionately affects individuals.^3^

*APOE* has three primary haplotypes (ε2, ε3, and ε4) defined by two single nucleotide polymorphisms, rs429358 (chr19:44908684:T:C) and rs7412 (chr19:44908822:C:T), which introduce amino acid changes resulting in the ε2 (Cys 112, Cys 158), ε3 (Cys112, Arg 158), and ε4 (Arg 112, Arg158) isoforms.^1^ The genetic burden for Alzheimer's disease associated with each *APOE* haplotype varies with the local ancestry of the *APOE* locus and demographic factors.^1^ Mexico's complex genetic architecture presents regional differences, with southern and central states exhibiting a higher percentage of Indigenous American ancestry compared with the north.^4^

Given recent efforts to investigate the genetic landscape of the Mexican population, we seized the opportunity to explore the genetic and epidemiological aspects of *APOE* in Mexico. In this study, we analyzed *APOE* haplotype frequencies in a large Mexican cohort (*n* = 6010) from the Mexican Biobank Project^4^ to investigate their regional variations and ancestry backgrounds across Mexico's 32 states.

*APOE* haplotype frequencies (ε2, ε3, and ε4) were calculated based on single nucleotide polymorphisms rs429358 and rs7412, with rs7412 imputed using TopMed. The single nucleotide polymorphism call rate of rs429358 was 99.53%, and after imputation, the r2 of rs7412 was 0.99111, reflecting a highly accurate imputation. The national haplotype frequencies were: ε3 at 0.876 (the most prevalent), ε4 at 0.1008, and ε2 at 0.024. Genotype analysis revealed that 4627 individuals were homozygous for ε3/ε3, 70 for ε4/ε4, and only 5 for ε2/ε2, the rarest genotype. Among heterozygotes, 1041 individuals carried the ε3/ε4 genotype, 236 had ε2/ε3, and 31 exhibited the ε2/ε4 combination.

The regional analysis found distinct patterns in haplotype distributions. ε4 frequencies ranged from 0.074 in Querétaro to 0.197 in Sonora, with the highest values observed in northern states, including Sinaloa, Tamaulipas, Durango, and Baja California. ε2 frequencies ranged from 0.0042 in Chiapas to 0.0586 in Aguascalientes, displaying a general north-to-south gradient. Across all regions, ε3 remained the predominant haplotype, with the lowest frequency in Sonora (76.97%) and the highest in Puebla (90.76%) (Fig. 1A).

**Figure 1:**
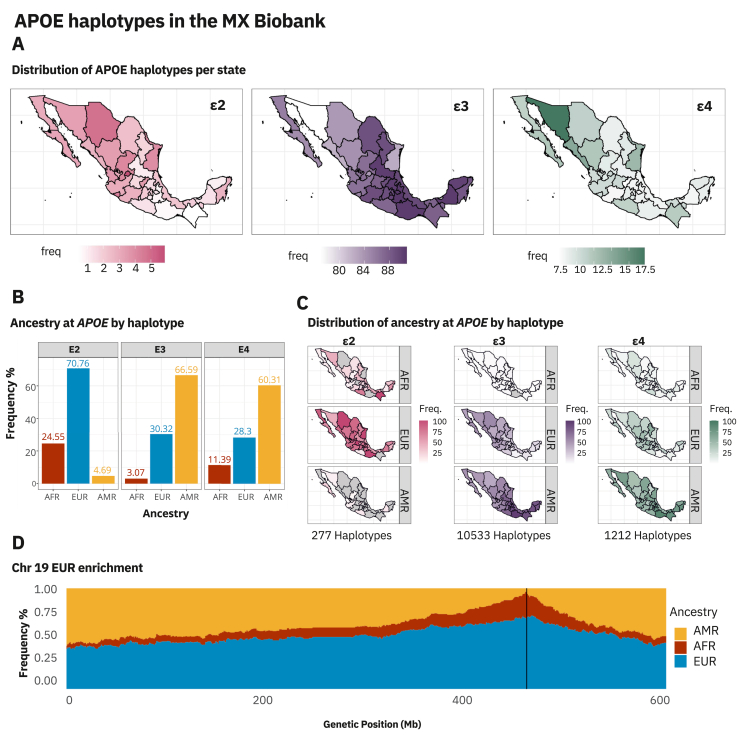
APOE haplotypes in the Mexican Biobank. **(A)** Map of *APOE* allelic frequency distribution across all Mexican states. The panel illustrates the distribution of *APOE* allelic frequencies across 32 Mexican states. The frequency scale is adjusted by the number of participants per state. It varies among haplotypes to highlight disparities in frequency haplotypes showing differences in frequency within the same haplotype map. *APOE* ε2 (pink), *APOE* ε3 (violet), and *APOE* ε4 (green). **(B)** Ancestry at *APOE* by haplotype. This plot presents three histograms, each showing the frequency of *APOE* ε2, ε3, and ε4 haplotypes categorized by African (AFR), European (EUR), or Indigenous American (AMR) ancestry. The *y*-axis represents frequency in percentage (per haplotype group), while the *x*-axis displays the three possible ancestries for each haplotype. *APOE* ε4 and *APOE* ε3 exhibit a significant AMR component (over 60%), while *APOE* ε2 shows a strong EUR frequency, followed by AFR (24.55%) and a minimal AMR component (4.69%). **(C)** Ancestry distribution across states by haplotype. The map panel displays the distribution of *APOE* ε2, ε3, and ε4 haplotypes across states, separated by ancestry (AFR, AMR, and EUR). The frequency scale is given in the number of participants per state and varies among haplotypes but remains the same within groups of maps (per haplotype), allowing identification of frequency differences. States colored in grey have no haplotypes reported for the ancestry/haplotype indicated. The scale of color ranges from the minimum haplotype frequency (lightest shade) to the highest haplotype frequency (darkest shade). **(D)** Enrichment of European Ancestry on Haplotype ε2. The plot depicts ancestry percentages across chromosome 19 in our sample. The *y*-axis shows the frequency of each locus categorized as AFR, EUR, or AMR. At the same time, the *x*-axis displays the position of each locus by its base pair within chromosome 19. A black line marks the *APOE* locus next to the 400 base pair label. The plot illustrates a significantly higher European ancestry percentage at the *APOE* locus for *APOE* ε2 carriers compared with adjacent areas on chromosome 19.

To further investigate the ancestral background of *APOE* haplotypes, we inferred local ancestry in the haplotype-defining region using Gnomix. Reference populations included European (EUR), African (AFR), and Indigenous American (AMR) samples from the 1000 Genomes Project, supplemented by 50 Mexican Biobank Project samples with >95% Indigenous ancestry. Each *APOE* haplotype (ε2, ε3, and ε4) was assigned an inferred ancestry, and haplotype frequencies were segmented by ancestry group. Homozygous individuals contributed both haplotypes to a single ancestry-haplotype pool, while heterozygous individuals contributed one haplotype to each relevant ancestry-haplotype pool (Table S1).

Our findings revealed that across all *APOE* haplotypes, the ancestry composition was predominantly AMR (64.53%), followed by EUR (31.05%) and AFR (4.4%). Analysis of each haplotype revealed distinct ancestry patterns: ε2 was primarily of EUR origin (70.7%), with contributions from AFR (24.55%) and minimal AMR (4.69%); ε4 had a strong AMR component (60.3%), with EUR (28.3%) and AFR (11.39%) contributions; and ε3, the most common haplotype, was predominantly of AMR origin (66.5%), followed by EUR (30.3%) and AFR (3.07%) (Fig. 1B).

Regionally, ε2 of EUR origin was more frequent in the north–central and northern states, particularly in Aguascalientes (5.8%) and Chihuahua (4.8%), while AMR-associated ε3 and ε4 haplotypes were more common in southern states, consistent with known geographic patterns of AMR ancestry within Mexico (Fig. 1C). This trend reinforces previous findings that highlight a higher prevalence of Indigenous ancestry in southern regions and higher EUR ancestry in the north.^4^

To contextualize the relevance of the differential distribution of APOE haplotypes across Mexico, we analyzed the incidence of Alzheimer's disease using data from the Mexican Epidemiology Surveillance System (SUIVE, part of the Ministry of Health). Specifically, we utilized records from the Anuario de Morbilidad (1984–2022) provided by the Dirección General de Epidemiología (https://epidemiologia.salud.gob.mx/anuario/html/index.html; accessed July 22, 2024). The population was grouped into three main age brackets (50–59, 60–64, and 65+), and the incidence of Alzheimer's disease per age group was standardized by accounting for the nationwide proportion of individuals in each category for every state.

Our analysis revealed that Colima had the highest incidence rate, with 30 cases per 100,000 inhabitants, followed by Sinaloa (10 cases per 100,000), Tamaulipas (9 cases per 100,000), Chihuahua (9 cases per 100,000), Baja California (7 cases per 100,000), and Coahuila (7 cases per 100,000), all of which are northern states. This aligns with the observed distribution of the *APOE*4 haplotype, which shows a north-to-south gradient. In contrast, states with the lowest incidence rates, at approximately one case per 100,000 inhabitants, included Guanajuato, Puebla, Zacatecas, Baja California Sur, and Mexico City.

To further elucidate the ancestral origin of ε2 haplotypes within the Mexican population, we conducted ancestry enrichment analysis along chromosome 19. This analysis revealed a notably higher EUR component within the *APOE* locus among ε2 carriers compared with adjacent chromosomal regions (Fig. 1D).

This study provides valuable insights into the frequency and ancestry background of *APOE* haplotypes in a representative Mexican cohort. Previous studies, including the Hispanic Community Health Study of Latinos, assessed over 10,000 Latino samples, including approximately 3600 self-reported Mexicans, revealing frequencies of 0.862 for ε3, 0.028 for ε2, and 0.11 for ε4,^3^ patterns largely in line with our findings. In their Mexican cohort, ε2 and ε3 haplotypes (0.023 and 0.876, respectively) differ from those observed in Europeans (0.08 and 0.78, respectively).^2^ Additionally, the ε4 frequency in our cohort (0.1008) is lower than the EUR average (0.14).^2^

Our results highlight a unique geographical distribution of *APOE* haplotypes in Mexico. The northern states exhibit the highest frequencies of ε4, the predominant risk factor for late-onset Alzheimer's disease,^2^ while the distribution of ε2 mirrors this pattern, despite ε2 being of predominantly EUR ancestry (over 70%). This aligns with the generally higher EUR ancestry in northern Mexico.^4^ A study by Ojeda-Granados et al^5^ found similar patterns, with high ε4 prevalence among Indigenous Huichol individuals from Nayarit (29%) and low ε2 frequencies among the Indigenous populations examined. Admixed individuals in the same study exhibited an ε2 frequency of 4.6%. Although this prior study focused on a small sample from specific regions, its findings corroborate our results and suggest that *APOE* ε2 is introduced through European colonization, whereas *APOE* ε4 has pre-Hispanic origins.

Our study is notable for its robust sampling and representation, covering not only urban areas but also rural communities and Indigenous populations throughout Mexico. This is the first nationally representative study of *APOE* haplotypes and ancestry in Mexico, with extensive participant coverage. Future research should examine the specific effects of *APOE* haplotypes on Alzheimer's disease risk and the interaction between these haplotypes and Mexico's unique genetic background.

In conclusion, this study characterizes the frequency, geographic distribution, and ancestral origins of *APOE* haplotypes — the most significant genetic risk factor for Alzheimer's disease — in a representative Mexican cohort. We identified specific geographic regions of particular interest due to their distinct frequency patterns of the *APOE* ε4 haplotype, higher incidence of Alzheimer's disease, and connections to Mexico's colonial and migratory history. These findings underscore the complex genetic landscape of Alzheimer's disease risk in Mexico and highlight the importance of considering ancestry and regional differences in genetic studies of Alzheimer's disease.

## Ethics declaration

The research team obtained written informed consent from all participants, and the project was conducted with approvals and under the oversight of the Research Ethics Committee and the Biosafety Committee of the Instituto Nacional de Salud Pública (Institutional Review Board approvals CI: 1479 and CB: 1470).

## Funding

M.E.R. thanks support from the 10.13039/501100001061Rebecca L. Cooper Medical Research Foundation (No. F20231230) and a Pilot Award for Global Brain Health Leaders by the Global Brain Health Institute, Alzheimer's Association, and Alzheimer's Society (GBHI ALZ UK-22-869,020). V.F.-O. is a recipient of a scholarship from the GP2 Trainee Network, part of the Global Parkinson's Genetics Program, and funded by the 10.13039/100018231Aligning Science Across Parkinson's initiative. The Mexican Biobank Project was supported by Mexico's National Council of Humanities Science and Technology (CONAHCYT; No. FONCICYT/50/2016), and The Newton Fund through the 10.13039/501100000265UK Medical Research Council (No. MR/N028937/1) awarded to A.M.-E. M.T.T.-L receives funding from PAPIIT-UNAM (No. IG200421). J.S.Y. receives funding from the NIH-NIA R01AG062588, R01AG057234, P30AG062422, P01AG019724, U19AG079774; NIH-NINDS U54NS123985; NIH-NIDA 75N95022C00031; the Rainwater Charitable Foundation; the Bluefield Project to Cure Frontotemporal Dementia; the Alzheimer's Association; the Global Brain Health Institute; the French Foundation; and the Mary Oakley Foundation.

## CRediT authorship contribution statement

**Carmina Barberena-Jonas:** Writing – original draft, Visualization, Methodology, Investigation, Data curation. **Victor Flores-Ocampo:** Writing – original draft, Investigation. **Natalia S. Ogonowski:** Writing – review & editing, Supervision. **Stefanie Danielle Piña-Escudero:** Writing – review & editing. **Ignacio F. Mata:** Writing – review & editing. **Jennifer S. Yokoyama:** Writing – review & editing. **Lourdes García-García:** Writing – review & editing. **Carlos Alberto Aguilar Salinas:** Writing – review & editing. **María Teresa Tusié-Luna:** Writing – review & editing. **Andrés Moreno-Estrada:** Writing – review & editing, Resources, Methodology, Data curation. **Miguel E. Rentería:** Writing – original draft, Supervision, Resources, Methodology, Funding acquisition, Conceptualization.

## Conflict of interests

J.S.Y. serves on the scientific advisory board for the Epstein Family Alzheimer's Research Collaboration.
