# Supplementary material for: Genetic analysis of APOE reveals distinct origins and distribution of ancestry-enrichment haplotypes in the Mexican Biobank
Source: Genes Dis. 2025 Jan 22;13(1):101542. doi: 10.1016/j.gendis.2025.101542 (PMC12495275; doi:10.1016/j.gendis.2025.101542)
Supplement: Multimedia component 1 [file mmc1.docx]

**Supplementary methods and results**

**Genetic Analysis of APOE Reveals Distinct Origins and Distribution of Ancestry-Enrichment Haplotypes in the MX Biobank**

**METHODS**

**Mexican cohort samples**

The Mexican Biobank Project (MXB) [^1^](https://paperpile.com/c/4rvlwd/2D6Hk) recruited 6010 participants across 32 Mexican states. MXB aims to provide insights into the Mexican population's genetic architecture and address the issue of the underrepresentation of Mexicans and Latinos in large-scale genetic studies. A detailed description of MXB recruitment is reported elsewhere [^1^](https://paperpile.com/c/4rvlwd/2D6Hk).

The MXB included participants who (i) participated in the Mexican National Health Survey (Encuesta Nacional de Salud [ENSA 2000]) [^2^](https://paperpile.com/c/4rvlwd/7ooG), (ii) were aged 20 years or older, and (iii) resided in Mexico at the time of the survey. Particular emphasis was placed on remote and rural regions, covering various phenotypes. The research team obtained written informed consent from all participants, and the project was conducted with approvals and under the oversight of the Research Ethics Committee and the Biosafety Committee of the Instituto Nacional de Salud Pública (INSP). Detailed information regarding the assessments and research methodology utilised in the ENSA study can be found elsewhere (Institutional Review Board approvals CI: 1479 and CB: 1470) [^1,2^](https://paperpile.com/c/4rvlwd/7ooG+2D6Hk).

**Cohort genotyping and quality control**

DNA was extracted from blood samples obtained from participants. All samples used for this study underwent quality control and post-processing. Genotyping with Illumina’s Multi-Ethnic Global Array (MEGA) that contains information for over 1.7 million SNPs. Genotyping was performed at the Unidad de Genómica Avanzada of the Laboratorio Nacional de Genómica para la Diversidad (LANGEBIO) of the Centro de Investigación y Estudios Avanzados del IPN (Cinvestav) in Irapuato, México. Quality control included removing individuals and SNPs with > 5% missingness, as well as duplicate SNPs, monomorphic SNPs, and all SNPs with an ambiguous strand. Additionally, samples underwent phasing, and all related individuals were pruned for linkage disequilibrium [^1^](https://paperpile.com/c/4rvlwd/2D6Hk).

**Local Ancestry Inference**

**Reference panels for local ancestry**

The admixed nature of the Mexican population indicates that three main continental groups are the most appropriate ancestral references: European (EUR), African (AMR), and Native American (AMR) [^1^](https://paperpile.com/c/4rvlwd/2D6Hk). Reference samples were primarily sourced from the 1000 Genomes Project dataset [^3^](https://paperpile.com/c/4rvlwd/3x8Hl). The EUR reference group comprised 198 individuals from Iberian Spanish (IBS) and British in England and Scotland (GBR) backgrounds. We included 108 individuals from Yoruba in Ibadan, Nigeria (YRI), from the AFR reference. Additionally, the AMR reference panel consisted of 79 individuals from Peru, Lima (PEL), predominantly of Native American ancestry, supplemented by 50 samples from MXB individuals, each exhibiting over 95% Indigenous Ancestry.

**Local ancestry analyses**

We conducted a local ancestry analysis on the genotyping data using Gnomix [^4^](https://paperpile.com/c/4rvlwd/x5FfM), a versatile tool that employs a modular framework. Gnomix estimates ancestry probabilities within genomic windows and then combines and refines these estimates to enhance the labelling accuracy, leveraging external ancestry references [^4^](https://paperpile.com/c/4rvlwd/x5FfM).

Gnomix relies on a “model” reference encompassing all sample references included in the panel to make ancestry inferences. Subsequently, the tool employs this model with our target sample, the “query”, to infer an ancestry label for each SNP common to both datasets, aided by the available phasing information.

Initially, we identified the intersection of SNPs of interest between the reference panel described previously and our query file, the MXB sample, focusing on chromosome 19. The intersected panel was formatted to include labels for each sample’s ancestry to convert it into a model that could be used with Gnomix. Finally, both the model and the query file, along with the specification of chromosome number (chr 19), were fed into Gnomix to generate its output. The output includes re-phasing information for the query, multiple statistical metrics, and per-SNP local ancestry labels.

**Allele frequency**

SNP rs429358 was directly genotyped, while rs7412, the second SNP defining the *APOE* haplotype, was not included in the MEGA array. We used TopMed for imputation to include rs7412; this is the same process as other SNPs first included in the MXB [^1^](https://paperpile.com/c/4rvlwd/2D6Hk)**.**

We calculated allele frequency for the three *APOE* haplotypes using the two defining SNPs: rs429358 and rs7412. The ε2 haplotype is defined by having T at both SNPs, ε3 by having T at rs429358 and C at rs7412, and ε4 by having C at both SNPs. Subsequently, counts for haplotypes were made using an in-house script. Additionally, we performed a directed allele frequency calculation, utilising the local ancestry of the *APOE* locus of each allele from each individual as a categorisation parameter, as described above.

We determined the percentage of haplotypes corresponding to each ancestral reference, irrespective of the allele they encoded. Furthermore, we assessed the frequency of *APOE* haplotypes labelled under one of the three references—*APOE* ε2, *APOE* ε3, and *APOE* ε4 —segregating them by allele group. This implies that a single individual can contribute to at least one frequency pool if homozygous or, at most, two frequency pools if heterozygous.

**Alzheimer’s Disease incidence in Mexico**

To describe the incidence of AD per state in Mexico, we consulted the Mexican Epidemiology Surveillance System (SUIVE, for its acronym in Spanish, part of the Health Secretary) [^5^](https://paperpile.com/c/4rvlwd/HIDTE) for the number of new AD cases reported per state yearly, in addition to the number of inhabitants per state, stratified by age group, collected by the National Population Council (CONAPO, for its acronym in Spanish) [^5^](https://paperpile.com/c/4rvlwd/HIDTE).

To calculate a standardised incidence rate, we adjusted the incidence by age. To achieve this, we grouped the number of AD cases and inhabitants in three age groups (50-59, 60-64, 65+). Subsequently, we calculated the incidence of AD per state for each age group separately. Finally, we multiplied the incidence rate of each state’s age group by the nationwide proportion of people in the same age group category.

**RESULTS**

**MXB cohort**

A total of 6,010 subjects (4,186 women) participated in the study. The age distribution of the subjects at the time of enrollment was 55% (20-39 years), 30% (40-59 years), and 15% (>60 years).

Analysis of the variants revealed 4,627 participants were homozygous for the *APOE* ε3 allele, while 70 individuals exhibited homozygosity for *APOE* ε4. The *APOE* ε2 homozygous genotype was found in only five individuals, representing the rarest genotype in our cohort. Among heterozygous combinations, 1,041 individuals showed *APOE* ε4 and *APOE* ε3 alleles, while 236 participants carried a combination of *APOE* ε2 and *APOE* ε3 alleles. Additionally, 31 individuals showed a heterozygous combination of *APOE* ε2 and *APOE* ε4 alleles (Supplementary Table 1).

**Geographical distribution of *APOE* haplotypes across Mexico**

The allele frequencies at the national level were 0.876 for the *APOE* ε3 haplotype, 0.10 for *APOE* ε4, and 0.024 for *APOE* ε2. We observed variations in the frequency of the *APOE* ε4 allele across Mexico’s 32 states, ranging from 0.074 in Queretaro, situated in Central Mexico, to 0.197 in Sonora, the northernmost state. Northern states had the highest *APOE* ε4 frequencies. The top 5 states with the highest *APOE* ε4 frequencies included Sonora, Sinaloa, Tamaulipas, Durango, and Baja California.

The *APOE* ε2 allele frequency ranged from 0.0042 in Chiapas to 0.0586 in Aguascalientes. Similar to the *APOE* ε4 distribution, states in the northern and western regions also had the highest *APOE* ε2 frequencies, including Chihuahua, Colima, Zacatecas, Tamaulipas, and Nayarit (Figure Panel A). *APOE* ε2 frequency follows a gradient, with higher frequencies in the country's north and then fades going south, similarly to *APOE* ε4 frequencies (Figure Panel A).

The distribution of the *APOE* ε3 across the 32 Mexican states ranged from 76.97% in Sonora to 90.76% in Puebla, emerging as the predominant most common allele in Mexico, with no discernible geographic patterns being observed (Figure Panel A).

**Geographical Variation of *APOE* Haplotypes by Ancestry**

Overall, 64.53% of *APOE* alleles had an AMR ancestry origin, 31.05% were EUR, and 4.4% were AFR. The majority of *APOE* ε2 haplotypes were labelled as EUR (70.76%), followed by AFR (24.55%) and AMR (4.69%). Conversely, the majority of *APOE* ε3 haplotypes were labelled as AMR (66.5%), followed by EUR (30.3%) and AFR (3.07%). Finally, the *APOE* ε4 haplotype exhibited a predominantly AMR ancestry (60.3%), followed by EUR (28.3%) and AFR (11.39%) (Figure Panel B).

*APOE* ε2 AFR haplotypes showed an uneven representation across Mexico due to its overall low prevalence and complete absence in several states. Conversely, the *APOE* ε2 EUR haplotypes, the most common within the *APOE* ε2 group, had the highest representation across states. The northern region holds the highest frequencies for *APOE* ε2 EUR, with Aguascalientes (5.8%) and Chihuahua heading the list (4.8%). Similarly to *APOE* ε2 AFR, *APOE* ε2 AMR haplotypes are under-represented in most states (Figure Panel C).

The AFR haplotype had the lowest representation of *APOE* ε3 across the country in our sample, being completely absent in several states. On the other hand, *APOE* ε3 EUR showed a north-to-south (i.e., higher-to-lower) trend. Conversely, *APOE* ε3 AMR follows the opposite trend and has higher frequencies in southern states (Figure Panel C).

We observed a poor representation of the *APOE* ε4 AFR haplotypes across the country without a clear pattern. *APOE* ε4 EUR haplotypes, however, showed a subtle north-to-south (i.e., higher to lower) trend across states. *APOE* ε4 AMR had the highest representation within the *APOE* ε4 group in Mexico. Moreover, the Southern states had the highest *APOE* ε4 AMR frequencies (Figure Panel C ).

We further analysed the ancestry of the *APOE* ε2 haplotypes along the whole chromosome 19 to elucidate the ancestral origin of this allele within the Mexican population. Interestingly, the *APOE* locus for the *APOE* ε2 haplotype carriers displayed a significantly higher percentage of EUR ancestry than nearby regions within the same chromosome. Moreover, both rs429358 and rs7412 received the same ancestry classification per individual across all samples, facilitating the assignment of ancestry labels to each participant’s haplotypes and enabling the analysis of ancestry frequencies per allele (Figure Panel D).

**Alzheimer’s Disease Incidence in Mexico**

We obtained the latest demographic information for the number of yearly new cases of AD in Mexico per state and age group (2022 SUIVE census) [^5^](https://paperpile.com/c/4rvlwd/HIDTE) and the number of inhabitants per state and age group (2022 SUIVE-CONAPO) [^5^](https://paperpile.com/c/4rvlwd/HIDTE). After standarising the incidence of AD for age, we observed that Colima held the highest incidence rate of 30 people for every 100,000 inhabitants, followed by Sinaloa (10 cases per 100K individuals), Tamaulipas (9 cases per 100K individuals), Chihuahua (9 cases per 100K individuals), Baja California (7 cases per 100K individuals) and Coahuila (7 cases per 100K individuals), all Northern states. States with the lowest incidence rate (for about one person for every 100,000 individuals) included Guanajuato, Puebla, Zacatecas, Baja California Sur, and CDMX.

**REFERENCES**

1. [Sohail M, Palma-Martínez MJ, Chong AY, et al. Mexican Biobank advances population and medical genomics of diverse ancestries. *Nature*. 2023;622(7984):775-783. doi:](http://paperpile.com/b/4rvlwd/2D6Hk)[10.1038/s41586-023-06560-0](http://dx.doi.org/10.1038/s41586-023-06560-0)

2. [Sepúlveda J, Tapia-Conyer R, Velásquez O, et al. Diseño y metodología de la Encuesta Nacional de Salud 2000. *Salud Publica Mex*. 2007;49:S427-S432.](http://paperpile.com/b/4rvlwd/7ooG) <https://www.scielosp.org/article/spm/2007.v49suppl3/s427-s432/es/>

3. [A global reference for human genetic variation. *Nature*. 2015;526(7571):68-74. doi:](http://paperpile.com/b/4rvlwd/3x8Hl)[10.1038/nature15393](http://dx.doi.org/10.1038/nature15393)

4. [Hilmarsson H, Kumar AS, Rastogi R, Bustamante CD, Montserrat DM, Ioannidis AG. High Resolution Ancestry Deconvolution for Next Generation Genomic Data. *bioRxiv*. Published online September 21, 2021:2021.09.19.460980. doi:](http://paperpile.com/b/4rvlwd/x5FfM)[10.1101/2021.09.19.460980](http://dx.doi.org/10.1101/2021.09.19.460980)

5. [Anuario de Morbilidad 1984 -2022. Dirección General de Epidemiología. Accessed July 22, 2024.](http://paperpile.com/b/4rvlwd/HIDTE) <https://epidemiologia.salud.gob.mx/anuario/html/index.html>
